# Early Onset Dystonia: Complaints about Executive Functioning, Depression and Anxiety

**DOI:** 10.3390/brainsci13020236

**Published:** 2023-01-30

**Authors:** Maraike A. Coenen, Hendriekje Eggink, A. M. Madelein van der Stouwe, Jacoba M. Spikman, Marina A. J. Tijssen

**Affiliations:** Department of Neurology, University Medical Center Groningen, University of Groningen, 9700 GZ Groningen, The Netherlands

**Keywords:** early onset dystonia, behavior regulation, anxiety, depression, social functioning

## Abstract

Early Onset Dystonia (EOD) is thought to result from basal ganglia dysfunction, structures also involved in non-motor functions, like regulation of behavior, mood and anxiety. Problems in these domains have been found in proxy-reports but not yet in self-reports of EOD patients. The main questions are whether proxy-reports differ from those of patients and how problems relate to everyday social functioning. Subjective complaints about executive problems (BRIEF) and symptoms of depression and anxiety (CBCL) were obtained through a cross-sectional questionnaire study conducted on 45 EOD patients. Scores were in the normal range in patients and proxies. Proxy-rated behavior regulation was correlated with the estimated number of friends and quality of relations. Proxy-reported scores of depression correlated with the quality of relations and were higher than self-reports of adolescent/young adult patients. EOD patients and proxies do not seem to experience problematic regulation of behavior, mood and anxiety. Still, our study revealed two important aspects: (1) all measures were related to the estimated quality of relations with others, relating questionnaires to everyday social functioning; (2) proxies reported more symptoms of depression than patients. This may indicate overestimation by proxies or higher sensitivity of proxies to these symptoms, implying underestimation of problems by patients.

## 1. Introduction

Early Onset Dystonia (EOD) is a hyperkinetic movement disorder characterized by involuntary movement and/or abnormal postures, developing before the age of 26 years [1]. Dystonia is thought to result from basal ganglia network dysfunction [2]. The basal ganglia are connected to, among others, the dorsolateral prefrontal- and the lateral orbitofrontal cortex, the hippocampus, limbic- and paralimbic cortices [3,4]. Based on these connections, non-motor symptoms can be expected in dystonia patients.

Indeed, non-motor symptoms, such as pain, sleep problems, cognitive deficits and psychiatric problems, have been found in dystonia patients [5,6,7,8,9,10]. Research in adult and childhood dystonia patients has shown that these non-motor symptoms predict health-related quality of live (HR-QoL) even more than motor symptoms do [7,11,12,13,14,15]. In children, especially pain, mood, and executive functioning have been found to relate to HR-QoL [12]. Part of executive functioning is the regulation of behavior and emotions. Regulation of behavior and affect, especially mood and anxiety, heavily relies on the orbitofrontal and dorsolateral prefrontal cortex, involved in the basal ganglia network [16].

In adult dystonia patients, both executive functioning deficits [6,7] and dysregulation of mood and anxiety have been found, with studies showing a higher prevalence of depression and anxiety compared to controls [8,9]. Psychiatric disorders have also recently been found for children with EOD in questionnaires completed by parents [17]. Anxiety disorders, disruptive disorders, and to a lesser extent, depression seem to be the most common psychiatric disorders in children with movement disorders, such as dystonia. Yet, the consequences of psychiatric disorders with regard to social functioning in daily life have not been studied. Furthermore, these findings are based on the parent’s perspective, leaving the children’s perspective unknown. Data on executive functions in EOD patients are very scarce [18], and their role in EOD remains largely elusive.

In the present cross-sectional study, we aim to investigate complaints about the regulation of behavior and mood and relate these to social functioning in daily life. We include the patients’ perspective by asking them to fill out self-report questionnaires in addition to parent/proxy-rated questionnaires. Our main hypothesis is that complaints about the regulation of behavior and mood exist in EOD patients and that these are related to social functioning in daily life. In an exploratory manner, we also investigate possible differences between patients’ and parents’ perspectives.

## 2. Materials and Methods

### 2.1. Participants

The sample consisted of patients with (1) EOD diagnosed by a movement disorders specialist and (2) aged between 5 and 26 years at time of measurement. Children’s age ranged from 5 to 12 years. Patients were recruited from the movement disorders outpatient clinic of the University Medical Center Groningen. For children younger than 11, no self-report versions of the questionnaires exist. Therefore we used only proxy-reports for this age group. To facilitate interpretation of the results, we only analyzed data from patients with complete datasets. Reason for incomplete dataset was that parents found the items not applicable to their child. For adolescents/young adults (age range 13–26), we used proxy- as well as self-report questionnaires.

### 2.2. Material

#### 2.2.1. Complaints about Behavior Regulation

Participants reported on behavior regulation on the Behavior Rating Inventory of Executive Functioning (BRIEF [19]) for children and adolescents (5–18 years) and the BRIEF-Adult (BRIEF-A) for adults (18–65 years). One of the resulting indices is the Behavior Regulation Index (BRIEF-BRI-self, BRIEF-BRI-proxy), a summary measure of the subscales Inhibition, Switching, Emotion regulation and (for adults) Self-Monitoring. Test-retest reliability for the BRI is 0.95 over 6 weeks for the BRIEF-proxy and 0.77 over two weeks for the BRIEF-A-proxy. Test-retest reliability for the BRIEF-A-self is 0.73 over two weeks. The manual does not provide test-retest reliability for the self-report of the BRIEF. Construct validity was analyzed with a confirmatory factor analysis. This analysis supported the two-factor model for the BRIEF-proxy, BRIEF-A-proxy, BRIEF-self, and BRIEF-A-self.

Normative data are available for the Netherlands and are corrected for age and gender.

#### 2.2.2. Complaints about Mood and Anxiety

Participants filled out the Achenbach System of Empirically Based Assessment (ASEBA [20,21]) to report problems regarding mood and anxiety. ASEBA includes questionnaires for children and adolescents from 6–18 years and questionnaires for adults (19–56 years). Self-report versions are available for children and adolescents (11–18 years; Youth Self Report (YSR)) and adults (Adult Self Report (ASR)). Children’s parents fill in the Child Behavior Checklist (CBCL), and adult patients’ proxies fill in the Adult Behavior Checklist (ABCL). The DSM-related subscales anxiety and depression were used (depression-self, depression-proxy, anxiety-self, anxiety-proxy). Test-retest reliability for the anxiety subscale is 0.51 over 24 months for the CBCL (0.77 over 1 week for the ABCL) and 0.46 over 7 months for the YSR (0.86 over 1 week for the ASR). For the depression subscale, a test-retest reliability of 0.64 was found for the CBCL (0.83 over 1 week for the ABCL) and 0.55 for the YSR (0.86 over 1 week for the ASR). Construct validity (correlation between CBCL and DSM checklist) was found to be 0.63 for the anxiety subscale and 0.63 for the depression subscale (correlations between ASR and SCL-90 0.77 for depression and 0.64 for anxiety). Normative data are available for the Netherlands and are corrected for age and gender.

#### 2.2.3. Indicators of Daily Life Social Functioning

Number of close friends and quality of relationships with others were used as indicators of daily life social functioning. Proxies provided an estimation of the number of close friends on the CBCL and ABCL on a three-point Likert scale: 0 = no close friends, 1 = one close friend, and 2 = 2 or more close friends. In addition, proxies estimated the quality of relationships with close friends for young adults (ABCL) and with siblings, friends and parents for children (CBCL). The mean of the answers to these questions was used for the analysis.

#### 2.2.4. Verbal Intelligence

As complex cognitive functions relate to global cognitive functioning, estimated verbal intelligence was included using three subscales (Similarities, Vocabulary, Digit Span) of the Wechsler Intelligence Scale for Children [22] or the Wechsler Adult Intelligence Scale [23].

#### 2.2.5. Motor Functioning

The Clinical Global Impression of severity (CGI-s) was used to score dystonia severity [24]. This score ranges from 1 (no dystonia) to 7 (most severe dystonia).

### 2.3. Procedure and Statistical Analysis

Patient data were obtained through regular patient care between 2013 and 2017 and processed anonymously. An exploratory analysis was performed, comparing patient scores to normative data. We compared proxy-reported scores of EOD patients with and without evidence of neuropathology for both age groups and proxy-data to self-reports in the group of adolescents/young adults. The raw scores were transformed into T-scores with a mean of T = 50, indicating normal functioning, and a standard deviation of 10. Clinically elevated scores are defined as scores 1.5 standard deviations above the mean.

Appropriate for non-normal distributions, product-moment correlation coefficients (*r* = z/(√N)), according to Rosenthal [25], were calculated as effect sizes and transformed into Cohen’s d. [26] Effect sizes were classified as *d* = 0.2 as small, *d* = 0.5 as moderate and *d* = 0.8 as large [27]. The significance level was α = 0.05 or adjusted (Bonferroni–Holm) in case of multiple comparisons. Missing data were addressed by casewise deletion.

The Medical Research Ethics Committee reviewed the study and declared the Medical Research Involving Human Subjects Act not applicable (MREC number 2013/012). Patients and proxies were informed about the use of their data for this study and did not object.

## 3. Results

Forty-five patients were included. The majority (*n* = 29) developed EOD within the first months of life (Table 1). Diagnoses varied between patients underlining the broad exploratory character of our study (Table 2). Drug treatment also varied in the group, but the majority of patients took no medication or were treated with central or peripheral muscle relaxants or trihexyphenidyl only (*n* = 27, see Table 3). Mean CGI-s scores were in the range of modest to distinct impairment (Table 1). Neither CGI-s scores nor disease duration was significantly correlated with parent- or patient-reported questionnaire scores. We chose to focus on nervous system pathology on the etiology axis in the most recent classification system [1]. There were 27 patients without evidence of neuropathology in our sample and 18 with such evidence.

Patients with evidence of neuropathology had significantly higher CGI-s scores compared to patients without evidence of neuropathology (Mann–Whitney U = 100, *p* = 0.001). There were no differences between these groups concerning age, IQ, or disease duration. Estimated verbal IQ was available for nine children and 26 adolescents/young adults (Table 1). For ten patients (five children, five adolescents/young adults), assessment of IQ was not possible because of their motor impairment. IQ scores did not differ between patients with and without evidence of neuropathology and were not significantly correlated with BRIEF-BRI scores or depression- or anxiety scores.

### 3.1. Reported Problems with Behavior Regulation

Median BRIEF-BRI-self and BRIEF-BRI-proxy scores were normal for the whole sample, as well as for children and adolescents/young adults, separately and did not differ between patients with and without neuropathology (Table 4). For adolescent/young adult patients, BRIEF scores did not differ between proxy- and self-reports (Table 5). Proxy-reported scores for three patients were in the clinical range, while only one scored that high.

### 3.2. Reported Symptoms of Depression and Anxiety

Median scores for depression-self, depression-proxy, anxiety-self, and anxiety-proxy were normal for the whole sample as well as for adolescent/young adult patients and did not differ between patients with and without neuropathology (Table 4). For children, depression-proxy and anxiety-proxy scores were on the border between normal and clinically elevated.

In the group of adolescents/young adults, depression-proxy and anxiety-proxy scores were higher than depression-self and anxiety-self scores, but after Bonferroni–Holm correction, only the difference between depression-proxy and depression-self remained significant with a large effect size (Table 5). This difference was not found for patients with and without neuropathology separately. Six proxies scored in the clinical range for depression, but only two of these patients had such high scores. Two additional patients scored in the clinical range, while their proxies reported normal scores. For symptoms of anxiety, two proxies scored in the clinical range, but none of the patients scored that high.

### 3.3. Relation with Indicators of Daily Life Social Functioning

BRIEF-BRI-proxy scores were significantly negatively correlated with the estimated number of friends (rho = −0.31, *p* = 0.05) and with the estimated quality of relationships (rho = −0.46, *p* = 0.002). Neither depression-proxy (rho = −0.068, *p* = 0.8) nor anxiety-proxy scores (rho = −0.30, *p* = 0.3) were significantly correlated with the estimated number of close friends. Both were, however, significantly negatively correlated with the estimated quality of relationships (depression: rho = −0.43, *p* = 0.005; anxiety: rho = −0.34, *p* = 0.03).

## 4. Discussion

By means of this cross-sectional questionnaire study, we explored whether children and adolescents/young adults with EOD and their proxies observe problems regarding the regulation of behavior, mood and anxiety. Overall, it appears that proxies observe and report more problems in these domains than patients themselves, but that average levels of reported problems are not within the clinically elevated range.

Contrary to our hypothesis, neither proxies nor patients themselves reported higher numbers of problems regarding behavior regulation or symptoms of depression and anxiety in comparison to ratings of problems in healthy peers. Also, there were no differences in the number of reported or observed problems between patients with and without evidence of neuropathology visible on an MRI scan. The finding that patients nor proxies report problematic behavior regulation, which is a relevant aspect of executive functioning, is not in line with the presumed role of basal ganglia in executive functions [5,6]. An explanation might be that patients with EOD, because of their motor problems, are less likely to independently encounter and perform daily life tasks that require complex executive skills and therefore are ignorant of their actual capacities. 

Furthermore, possible problems concerning behavior regulation may not be visible for their proxies. Proxies of nine patients did not fill in the BRIEF, indicating that the BRIEF items might not apply to the everyday life of severely affected patients. The high prevalence of disruptive behavior in children with non-tic movement disorders, found by Lorentzos et al. [17], underscores the possible problems with behavior regulation in EOD. Studies on adult dystonia patients have documented executive deficits using objective neuropsychological tests [5,6,7,8,9,10]. We deem it likely that standardized neuropsychological tests for different aspects of behavior regulation (executive functioning and social cognition) may have yielded deficits, in particular in the more severely affected group of patients with evidence of neuropathology. In future research, neuropsychological tests are required to investigate whether these patients have impairments in behavior regulation.

Patient- and proxy-reported symptoms of depression and anxiety were also in the normal range. Yet, in line with our hypothesis, we found evidence that proxies of adolescent/young adult EOD patients report more symptoms of depression than patients themselves. Differences between parent- and patient-reported data have also been found in various other groups, such as children with aggressive behavior, attention deficits, learning disabilities and mood problems [28], with mean correlations of 0.2 between patients and parents. However, most studies have found lower scores for depressive symptoms reported by parents unless the parent is depressed him/herself, in which case they report higher levels of depressive symptoms in their child [29,30]. There are four possible explanations for our findings: proxies might over-report symptoms of depression and anxiety in patients (1) because of the lack of a clear prognosis about the social and emotional development of EOD patients [31], or (2) because they might mistake lack of initiative and/or decreased expression of emotions for signs of depression, where these are actually problems of behavior regulation, (3) patients might have impaired self-insight, which is a common element of impaired behavior regulation, and therefore only their environment notices these problems [5,8,9], and (4) EOD patients do not have symptoms of depression and anxiety in their youth, but these symptoms might develop later in life. We agree with Lorentzos et al. [17] that it is important to screen for psychiatric disorders in EOD patients and would like to stress the necessity of including the patient’s perspective. As we know, depression occurs more frequently in adult patients with dystonia than in the general population [6,7]; it might be possible that the first subtle symptoms develop during adolescence and are first noticed by proxies. Longitudinal data are needed to investigate this question. Still, in our cross-sectional sample, disease duration was unrelated to the reported symptoms of depression and anxiety.

Concerning indicators of social functioning in daily life, higher proxy ratings of behavior regulation were negatively correlated with the estimated number of good friends, indicating that more behavior regulation problems are related to fewer friends. In addition, all three proxy-rated measures (behavior regulation, depression and anxiety) were negatively correlated to the estimated quality of relationships with others, indicating that more problems in these areas are related to poorer quality of relations with others. These are important findings as they show that questionnaire scores can be related to everyday social functioning in EOD patients and that psychiatric comorbidity has a profound impact on everyday social functioning.

Our results have to be viewed in light of the limitations of the study design. We included a broad range of patients from different age groups and with different etiologies and explored the subjective experiences of these patients and their proxies. We acknowledge that regulation of behavior, mood and anxiety is still developing in our patients and that particular problems concerning behavior regulation will manifest differently across age groups. However, we did use questionnaires developed for the specific age groups in our sample. Drug treatment varied in our sample, but about 36% of our sample did not use any medication, and another 33% only used trihexyphenidyl and/or central and/or peripheral muscle relaxants (BTX, Baclofen) or vitamin E. Side effects concerning these treatments’ regulation of mood and behavior are unlikely; therefore we expect that medication use has little to no effect on our results. However, we acknowledge the limitation that we have not included medication as a confounding variable in the analysis. Doing so would have left us with a much smaller sample size and, thus, smaller statistical power.

With this study, we investigated the regulation of behavior, mood and anxiety in EOD patients. The results aid us in further grasping non-motor symptoms in this patient group, and future studies should investigate different aspects of behavior regulation (executive functioning and social cognition) through objective neuropsychological tests, as it is likely that some of the patients will have impairments that are not tapped by subjective measures. The finding that proxies of adolescent/young adult patients report more symptoms of depression than patients themselves deserves more research attention. Especially since these scores are also related to lower estimated quality of relations to others, making proxy-reports highly valuable for clinical practice. Possible causes of proxy observed depressive phenomena, such as patients’ problems regarding initiative, insight and emotional expressions, need to be studied. For clinical practice, our findings underline the importance of including proxy- and patient data in the assessment of depression, as proxies might notice subtle depressive symptoms sooner than patients themselves, allowing timely treatment. If the higher proxy-reported scores of depression turn out to be an overestimation, psychoeducation of proxies might help them to correctly interpret the patients’ behavior. Either way, proxies are an invaluable source of information for clinicians who work with EOD patients. In general, non-motor symptoms can have a negative impact on the quality of life and add to the perceived burden of the disorder [7,11,12,13,14,15]. Hence, a systematic evaluation of behavior regulation, depression and anxiety should be part of the EOD diagnostics and patient care, preferably including objective neuropsychological test results as well as the judgment of proxies of the patient’s functioning.

## 5. Conclusions

In this study, there is no evidence for increased problems concerning the regulation of behavior, mood and anxiety as reported by proxies of patients with Early Onset Dystonia (EOD). However, proxies report more depressive symptoms than EOD patients themselves. Furthermore, subjective complaints of proxies of EOD patients about the regulation of behavior, mood and anxiety are related to the number of friends and quality of relations.

## Figures and Tables

**Table 1:** Demographic characteristics.

	No Evidence of Neuropathology	Evidence of Neuropathology	Total
Children			
N (n_male_)	10 (6)	4 (2)	14 (8)
M_Age_ (SD)	10.1 (1.6)	8.2 (1.4)	9.6 (1.8)
Age range	8.3–12.7	7.3–10.3	7.3–12.7
M_Yrs since onset_ (SD)	8.3 (3.6)	8.2 (1.4)	8.3 (3.1)
M_IQ_ (SD)	104.3 (6)	57 ^†^	99 (16.7)
M_CGI-s_ (SD)	4 (1)	6.25 (1)	4.6 (1.5)
M_close friends_ (SD)	2.4 (1.2)	0.8 (1)	1.9 (1.3)
M_Qualiy of relations_ (SD)	1.9 (0.5)	2.1 (0.4)	1.9 (0.5)
Adolescents/Young adults			
N (n_male_)	17 (12)	14 (4)	31 (16)
M_Age_ (SD)	18.9 (3.3)	19.1 (4.4)	19 (3.8)
Age range	13.5–24.8	13.5–26.8	13.5–26.8
M_Yrs since onset_ (SD)	14.4 (6.3)	17.9 (4.8)	16 (5.8)
M_IQ_ (SD)	89.5 (20.1)	85 (15.4)	87.8 (18.2)
M_CGI-s_ (SD)	3.6 (1.4)	4.9 (1.2)	4.2 (1.4)
M_close friends_ (SD)	1.9 (1.7)	2.2 (1.2)	2.0 (1.5)
M_Qualiy of relations_ (SD)	2.3 (0.6)	2.1 (0.5)	2.2 (0.6)

† Only one child in this group was able to complete the intelligence test. CGI-S: Clinical Global Impression of severity.

**Table 2:** Diagnoses in the sample.

No Evidence of Neuropathology		Evidence of Neuropathology	
Diagnosis	Count	Diagnosis	Count
Children			
Myoclonus dystonia DYT11 positive	3	CP	2
DYT11 negative	2	Metabolic disorder	1
DYT12 positive	1	ARX	1
Generalized dystonia	1		
Silver Russel syndrome	1		
Metabolic disorder	1		
Unknown cause	1		
Total	10		4
Adolescents/Young adults			
Myoclonus dystonia DYT11 positive	2	CP	12
DYT11 negative	5	ADEM	1
DYT6 positive	1	Tumor	1
Cervical dystonia	2		
Segmental dystonia	1		
AVED	2		
RELN mutation	2		
ACTB mutation	1		
Unknown cause	1		
Total	17		14

AVED: ataxia with vitamin E deficiency, RELN: reelin gene, ACTB: actin beta gene, CP: cerebral palsy, ARX: Aristaless-related homeobox gene, ADEM: Acute disseminated encephalomyelitis.

**Table 3:** Drug treatments in the sample.

Medication	Frequency
None	16
**Monotherapy**	**18**
Anticholinergic	7
BTX	3
Baclofen	1
Vitamin E	1
Gabapentin	1
Levodopa	2
Methylphenidate	1
Benzodiazepine	2
**Combination Therapy**	**11**
Anticholinergic + BTX	3
Baclofen_ + Benzodiazepine	3
Baclofen or BTX + Trihexyphenidyl + Benzodiazepine	3
Baclofen + BTX	1
Anticholinergic + Benzodiazepine	1

BTX: injections with botulinum toxin.

**Table 4:** Mean (sd) of patient- and proxy-rated questionnaire scores and comparison (Mann–Whitney U) between EOD patients with and without evidence of neuropathology.

Scale	Informant	Whole Sample	N	No Evidence of Neuropathology	N	Evidence of Neuropathology	N	Difference Evidence/No Evidence of Neuropathology	Cohen’s *d*
Children									
BRI	Parents	47 (10.8)	14	46 (10.9)	10	50 (11.4)	4	U = 16, *p* = 0.6	*d* = −0.1
Depression	Parents	64 (17.0)	14	65 (13.5)	10	52 (16.5)	4	U = 10, *p* = 0.2	*d* = −0.8
Anxiety	Parents	59 (13.8)	14	60 (16.0)	10	54.5 (9.3)	4	U = 13.5, *p* = 0.4	*d* = −0.5
Adolescents/young adults									
BRI	Proxies	47 (10.2)	31	47 (8.4)	17	48 (12.3)	14	U = 112, *p* = 0.8	*d* = −0.1
	Patients	46 (10.2)	31	45 (9.2)	17	47 (11.6)	14	U = 111, *p* = 0.8	*d* = −0.1
Depression	Proxies	55 (12.0)	31	55 (12.5)	17	57.0 (11.8)	14	U = 112.5, *p* = 0.8	*d* = −0.1
	Patients	52 (8.0)	31	51 (7.5)	17	52.0 (9.3)	14	U = 108.5, *p* = 0.7	*d* = −0.2
Anxiety	Proxies	54 (9.0)	31	53 (10.5)	17	56.0 (9.3)	14	U = 111.5, *p* = 0.8	*d* = −0.2
	Patients	51 (6.0)	31	50 (6.0)	17	51.0 (11.3)	14	U = 99.5, *p* = 0.4	*d* = −0.3

BRI: Behavior Regulation Index of the BRIEF questionnaire.

**Table 5:** Wilcoxon Signed Rank comparison between proxy- and self-reports for the whole adolescent/young adult group and patients with and without evidence of neuropathology.

Scale	Difference Patients/Proxies					
	Whole Sample	Cohen’s *d*	No Evidence of Neuropathology	Cohen’s *d*	Evidence of Neuropathology	Cohen’s *d*
BRI	Z = −0.7, *p* = 0.5	*d* = −0.2	Z = −0.2, *p* = 0.8	−0.2	Z = −0.9, *p* = 0.4	*d* = −0.2
Depression	Z = −2.3, *p* = 0.02 *	*d* = −0.9	Z = −1.6, *p* = 0.1	−0.8	Z = −1.5, *p* = 0.1	*d* = −0.9
Anxiety	Z = −2.4, *p* = 0.02	*d* = −0.9	Z = −1.8, *p* = 0.07	−1	Z = −1.5, *p* = 0.1	*d* = −0.9

BRI: Behavior Regulation Index of the BRIEF questionnaire. * significant after Bonferroni–Holm correction.

## Data Availability

Data are available upon reasonable request to the corresponding author.
